# White blood cell estimates correlate to measures of population and individual health in an endangered population of Marbled Murrelets (*Brachyramphus marmoratus*)

**DOI:** 10.3389/fvets.2025.1545905

**Published:** 2025-05-15

**Authors:** Kelsey Ryan, Lindsay J. Adrean, Matt G. Betts, Jonathan Dachenhaus, Jennifer Johns, Miranda Michlanski, S. Kim Nelson, Shannon Phelps, James W. Rivers, Daniel D. Roby, Ethan Woodis, Brianna R. Beechler

**Affiliations:** ^1^Carlson College of Veterinary Medicine, Oregon State University, Corvallis, OR, United States; ^2^American Bird Conservancy, The Plains, VA, United States; ^3^Department of Forest Engineering, Resources, and Management, Oregon State University, Corvallis, OR, United States; ^4^Department of Forest, Ecosystems and Society, Oregon State University, Corvallis, OR, United States; ^5^Department of Fisheries, Wildlife, and Conservation Sciences, Oregon State University, Corvallis, OR, United States

**Keywords:** Marbled Murrelet, seabird, wildlife, reference interval, white blood cell count, population health, hematology, clinical pathology

## Abstract

**Introduction:**

Estimated white blood cell (WBC) counts are a valuable tool for assessing individual and population health in wildlife and domestic animals due to their role in the response to environmental stressors and disease. These measures are infrequently used in the study of wild seabird species, despite their utility when used alongside other common health assays - such as infectious disease testing, body condition, and population monitoring efforts. The Marbled Murrelet (*Brachyramphus marmoratus*) is a seabird of conservation concern that is federally listed as threatened by the states of Oregon, Washington, and California, thus necessitating the evaluation of its physiological health.

**Methods:**

We evaluated the utility of estimated WBC counts as measures of health, asking whether counts changed by measures of individual health (i.e., sex, *L. marmoratii* hemoparasite burden, body condition index, and nesting propensity) and population health (i.e., changes in counts by year). We used blood smears collected from over 350 murrelets captured along the Oregon Coast between April and June of 2017-2019 and 2021-2022 to estimate total WBC and differential counts.

**Results:**

Estimated WBC counts were found to appear lower in years with more favorable ocean conditions, when nesting propensity was relatively high. Male murrelets, individuals less likely to nest, and individuals with greater *L. marmoratii* burden had significantly lower estimated WBC counts, whereas individuals with a lower body condition index had elevated estimated WBC counts.

**Discussion:**

These results demonstrate the utility of estimated WBC counts to further assess health at the individual and population levels in the study of species of heightened conservation concern and should be considered as an addition to research plans.

## 1 Introduction

Common methods of studying wild seabird species generally focus on the collection of nesting, survival, and morphometric data (e.g., body measurements and mass) to assess population health and trends, species behavior, and factors affecting recruitment (1–8). A method that is less frequently used to assess wild seabird population health is the field of hematology, the study of blood and its cellular characteristics (9, 10). Factors such as insufficient sample size, small populations, or difficulty in sampling may limit use of this method in wild species (11). Nevertheless, blood-based metrics can be an informative way to further assess health in wild seabird populations, due to the immune system activities of the individual white blood cell types. The five types of white blood cells found in birds are categorized in two ways. In the first, there are three types of granulocytes; the heterophil, which functions through phagocytosis of infectious agents in the acute inflammatory response; and the basophil and eosinophil, both of which function in the hypersensitivity response, with eosinophils also functioning in some parasitic infections. In the second, there are two types of mononuclear cells; the lymphocyte, which functions through directing the immune response in the body via the action of T cells and B cells; and the monocyte, which functions in phagocytosis and antigen presentation to lymphocytes (12). Variations in leukocyte number may be present due to environmental or physiologic stress; lymphopenia or relative decrease in lymphocyte count and heterophilia or relative increase in heterophil count, may be seen as part of the stress response in the body (13). The study of the quantity and quality of these cell types gives valuable insight as to the immune system response in the body caused by stress or disease.

White blood cell count reference intervals for wild species are valuable because they allow comparison and detection of changes in individual and population health over time. Establishment of reference intervals for wild species allows a deeper assessment of individual health in these settings. Comparing obtained cell counts from an individual to an established reference interval for the population allows for a deeper assessment of individual health alongside other parameters, including physical appearance. Additionally, concerning their utility in monitoring population health over time, changes in reference intervals for a given population may be detected if studied over time, allowing further investigation as to the cause of these changes and opportunity for response.

The Marbled Murrelet (*Brachyramphus marmoratus*, hereafter murrelet) is a small, non-migratory diving seabird in the auk family (Alcidae), occurring from Alaska to central California along the Pacific coast of North America (14, 15). The species is listed as threatened in California, Oregon, and Washington under the U.S. Endangered Species Act (16, 17), thereby making the murrelet a species of conservation concern throughout its range in the contiguous United States. Murrelets were once common throughout their range, but studies investigating abundance of murrelets have reported that populations have continued to decline annually across their range (18, 19). Recent reports suggest that at-sea abundance of murrelets appears to be decreasing in the northern part of their range and increasing in the southern part of their range; however, the cause of these trends is likely multifactorial (20, 21).

Murrelets have an unusual breeding strategy, which makes them challenging to study. They forage for schooling fish and invertebrates in nearshore (within 5 km) marine waters, and they nest arboreally within mature coastal forests with occasional nesting on the ground and on rock ledges in the northern part of their range (22–25). This species can fly long distances inland (>80 km) as it socializes, searches for nest sites, and travels to and from nesting areas (15, 26, 27), though factors such as poor ocean conditions can cause murrelets to travel great distances (>500 km) away from their terrestrial nesting habitat in their selection of adequate marine habitat (28). Murrelet nests have been historically difficult to locate due to their small body size and cryptic nesting behavior of the species, and placement of nests high in trees or on cliffs located in rugged terrain (29–31). Murrelet pairs select a nesting location and work together to incubate a single egg. The pair will trade off incubation duties approximately every 24 h, leaving one at the nest and the other to forage at sea (25, 32). Although marine factors such as prey availability and quality being impacted by shifts in climate over time or acute elevations in ocean temperatures seasonally have likely contributed to murrelet population declines (33–35), the major cause is thought to be sustained low recruitment resulting from the loss of quality nesting sites and high rates of nest failure from predation related to edge effects (16, 18, 20, 30, 36–40).

Recently, the Oregon population of murrelets was found to harbor a previously undocumented species of *Leucocytozoon* hemoparasite (*Leucocytozoon marmoratii*) that was detected with a prevalence of 62% for the population (41). Protozoans in the *Leucocytozoon* genus belong to the avian Haemosporida order of vector-borne parasites which are part of the Apicomplexa phylum. *Leucocytozoon* hemoparasites have been identified and described in a number of avian species, such as raptors, songbirds, and poultry; however, they have rarely been found in seabirds (42–49). Michlanski (41) found that increased parasite burden (described as the number of *L. marmoratii* detected per 100 white blood cells) was associated with a reduction in nesting propensity, suggesting that the burden of *L. marmoratii* may be affecting murrelet health. It has not been investigated whether *L. marmoratii* burden is associated with changes in white blood cell differential counts in murrelets, although in other species *Leucocytozoon* hemoparasites may have impacts on immunologic, physiologic, and reproductive health (43, 44, 47). Thus, investigation to assess any changes in white blood cell counts due to *L. marmoratii* burden in murrelets is important.

There are several studies concerning the population health of the murrelet, the majority of which are focused on the collection of nesting, survival, and morphometric data (19, 30, 50, 51). There are limited studies concerning blood-based measures to evaluate individual and population health for this species (9). This study sought to assess the utility of white blood cell count estimations alongside other commonly collected parameters included in population and individual health assessments: sex, body condition index, nesting propensity, and *L. marmoratii* parasite burden. We also sought to generate reference intervals for estimated total white blood cell and differential count data. The goals of this project were as follows: (1) To determine if estimated white blood cell counts change relative to changing environmental conditions or other stressors affecting this population; (2) To use estimated white blood cell counts alongside other covariates (i.e., sex, body condition index, nesting attempt, and *L. marmoratii* burden) to investigate relationships that may show the utility of white blood cell count estimations as an additional assay that can be used to assess population and individual health in murrelets and other seabird species. We did this by assessing whether estimated white blood cell counts correlated to measures of individual health (sex, body condition index, *L. marmoratii* burden), as well as whether counts covaried with environmental conditions that influenced nesting propensity; (3) Use estimated white blood cell counts from blood smears to construct white blood cell count reference intervals for the Oregon population to be used as an additional method of monitoring the population for changes over time, as well as in rehabilitation and medical environments to assess the health of an individual animal.

## 2 Materials and methods

Field data collection occurred from late April to early June during 2017–2019 and 2021–2022 as part of a large-scale, long-term study investigation of murrelet nesting ecology in the coastal forests of central Oregon led by researchers in the College of Forestry at Oregon State University. To do this, teams departed from Newport, Oregon and undertook at-sea captures overnight within nearshore areas 35 km to the north or south. Working from a large vessel, a small inflatable boat was offloaded at sea to search for birds with a high-powered spotlight, and a large dip net was used to capture birds (52). Immediately after capture, birds were moved into plastic transport containers and transported to the research vessel for assessment and processing. Once on the research vessel, a brief physical examination was performed by experienced personnel, assessing relative stress levels and overall body condition of each bird. Most birds were healthy enough to continue, but if a bird was undergoing severe stress (panting severely), it was released prior to sampling. If considered healthy enough to proceed then a uniquely numbered metal identification band was placed on one of the bird’s legs and each individual measured for body mass (± 1.0 g) and culmen length (± 0.1 mm). A small sample of blood from the medial metatarsal vein was collected, between 0.6–1.0 mL per bird based upon body mass, using a heparinized 3 milliliter syringe (BD Luer-Lok disposable) with a 27 or 25 gage butterfly catheter (Terumo Surflo winged-infusion sets). The samples were used to make blood smears for white blood cell count estimates and parasite assessment, and a drop from each sample was placed on a Whatman FTA card for DNA sexing, as murrelets are not sexually dimorphic in size and plumage. For individuals that weighed ≥200 g, a small VHF telemetry tag (model A4330, 2.5 g, Advanced Telemetry Systems, Isanti, MN, US) was attached to the upper back using a subcutaneous anchor (53). In 2019 only, tail-mounted VHF telemetry tags were employed on a small number of individuals (*n* = 7); however, this approach was ineffective and discontinued due to poor tag retention. All birds were released within 1 h of capture and within 1 km of their original capture location.

After release, radio-tagged birds were tracked by fixed wing aircraft and via 72 ground-based, fixed telemetry stations that were located every 2–3 km along the coast across the 135 km study area, stretching from Pacific City, Oregon southward to Florence, Oregon. Using both methods, birds were tracked on a near-daily basis and thus we were able to detect the unique inland movement patterns murrelets exhibit during incubation that signaled an active nest (29, 32). In the analysis we call this variable nesting propensity, which is a binomial variable indicating whether a bird attempted to nest or not.

The process to obtain estimates for white blood cell count data was accomplished through manual counting from blood smears collected from captured individual murrelets. Blood smears were made on the research vessel immediately following blood collection and allowed to air dry and then transferred to the OSU Oregon Veterinary Diagnostic Laboratory for analysis. Prepared slides were stained using a modified Wright stain for preservation and to highlight blood cells and their contents. 100-cell white blood cell differential counts were performed, and total white blood cell counts were estimated using a standard manual blood smear technique (54). The five types of white blood cells found in birds and identified in differential counting are the heterophil, lymphocyte, monocyte, basophil, and eosinophil. Two observers (JJ and SP) performed the hematology evaluation. When evaluating for estimated white blood cell counts as well as parasite presence and burden, all blood smears were analyzed blind with respect to the main factors analyzed in this study, including birds that were male vs. female, body condition index, or birds that attempted a nest vs. those that did not. Minimal inter-observer variation was confirmed by both observers via comparison of WBC estimated counts and differentials on 20 samples. Although it has been demonstrated that manual WBC estimated counts and differential counts determined from blood smears may have a wider coefficient of variation when compared to automated methods (55), due to sample size, remote location of blood sample collection, and availability of equipment, manual methods, and thus WBC count estimation, was determined to be adequate for this project. Blood smears are used as the standard method for estimating WBC counts for non-mammalian species in the Oregon State University Veterinary Diagnostic Laboratory.

Evaluation for *L. marmoratii* burden was completed using the same blood smear slides that were used for estimating white blood cell counts, using the methods described by Michlanski (41). Parasite burden was determined by scanning the monolayer of each blood smear and counting the number of *L. marmoratii* detected per 100 white blood cells. In individuals where no parasites were detected in the monolayer, the feathered edge was also scanned in an attempt to identify individuals with low burden.

Sex was determined molecularly from samples cut from the center of a blood spot on a Whatman FTA card which were used for the extraction of genomic DNA. Samples were stored at room temperature until time of analysis. Amplification of the chromo-helicase-DNA-binding protein genes of the Z and W chromosomes was completed using a standard method for DNA sexing of bird species (56). Each sample was amplified in a minimum of three independent PCR replicates to verify results. Female samples produced two bands at approximately 400 bp and 600 bp, while male samples showed only a 600 bp band.

To estimate body condition index (BCI), we calculated residual values from a regression of body mass to tarsus length in GraphPad Prism, with residuals representing each bird’s BCI, with positive values indicating better condition (i.e., larger body mass than expected body size) and negative values indicative of worse condition (i.e., smaller body mass than expected body size) Residuals can be confounded by sex due to variation in BCI between males and females (57, 58), so we performed linear analysis and calculated residuals separately for males and females given that some females were likely in the process of developing eggs (personal observations, BB) when captured, and given that the capture period of the study took place in the breeding to early nesting period for the species (32).

Generation of reference intervals were completed using the guidelines for veterinary species set forth by the American Society of Veterinary Clinical Pathologists (ASVCP) Quality Assurance and Laboratory Standards Committee, which advise standards on sample collection, methods for identifying outliers, and minimum sample size (11). Only samples from clinically healthy birds were included for reference intervals, based on a brief physical examination at time of capture. Upon initial assessment of the blood smear slides, we found that *L. marmoratii* were detected in 233 of the 374 birds that were sampled (41). Microfilariae were also observed in five samples. Uniquely numbered metal leg bands allowed identification of recaptured individuals, which occurred in eight instances; a randomly selected blood smear was used for individuals who were captured in multiple years. The omission of these samples, as well as samples where *L. marmoratii* and Microfilariae were detected, reserved 130 samples from which non-parametric reference intervals could be generated (11). We used all 5 sample years in the generation of the reference intervals to give a representative range of counts for a wild population. Reference Value Advisor, a macro instruction for Microsoft Excel was used to calculate reference limits (59). This test allowed for the detection of outliers using the Tukey and Dixon-Reed tests. Upon identification of potential outliers by the program, we determined through slide reassessment that these values may not be errors, but instead could be normal variation in white blood cell counts and were not removed.

Analyses evaluating correlations between white blood cell counts estimates and year, sex, body condition index, nesting status, and *Leucocytozoon* burden were completed using R and GraphPad Prism software. Eosinophils are uncommon in many avian species, which was found to be true for murrelets in this study. Therefore, we decided to not include eosinophils in further analyses. In comparing white blood cell counts by year, a Kruskal-Wallis ANOVA test with Dunn’s correction for multiple comparisons was completed in GraphPad Prism for each cell type. To assess whether sex, body condition index, parasite burden, or nesting propensity correlated to estimated white blood cell counts, we used a generalized linear model with quasi-Poisson distribution in R, accounting for year using the lme4 and lmer.test packages (60). The dependent variable for the model was each white blood cell count (one model each for total white blood cells count and the differential counts for the separate cell types, excluding eosinophils), and the independent variables were sex, body condition index, parasite burden, and nesting propensity. Independent variables that were not binomial (parasite burden and body condition index) were rescaled using the scale function in the MASS package in R to allow comparisons of the beta estimates from outputs (61).

## 3 Results

### 3.1 Interannual variation in murrelet white blood cell count estimates

**Figure 1:**
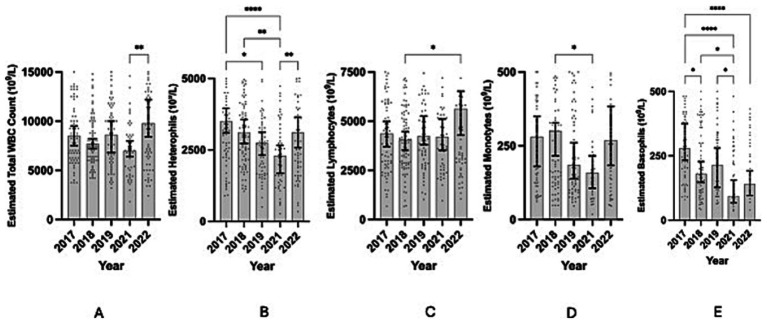
Scatter plots showing estimated white blood cell counts by cell type for each year that samples were collected during the project. Top of the shaded area reaches the sample median. Black error bars mark the 95% confidence interval for the estimated white blood cell count sample median for each year. Asterisks mark significance of the difference between different years with more numerous symbols corresponding to a more significant relationship (based upon a *p*-value of less than or equal to 0.05). **(A)** Estimated total white blood cell counts vs. year. **(B)** Estimated heterophil counts vs. year **(C)** Estimated lymphocyte counts vs. year **(D)** Estimated monocyte counts vs. year. **(E)** Estimated basophil counts vs. year. Y-axis scale is reduced to allow best visualization for each plot, where some data points are omitted in each plot as a result.

Estimated total white blood cell counts varied significantly among years (Kruskal-Wallis: *H* = 15.12, *p* = 0.0045); however, *post hoc* comparisons showed that the significant difference was primarily driven by differences between 2021 and 2022, while other pairwise comparisons were not significant (Figure 1A; Table 1). For each blood cell type, heterophils and basophils varied significantly between many years but lymphocytes and monocytes were more stable with the exception of one small difference each (Figure 1). Estimated heterophil counts varied significantly by year (Kruskal-Wallis ANOVA; *h* = 24.59, *p* < 0.0001) (Figure 1B). Differences by year were explained by mean rank counts for 2021 being significantly lower in comparison to several other years: 2017 (*p* < 0.0001), 2018 (*p* = 0.0066), and 2022 (*p* = 0.0092), and 2017 being significantly higher than 2019 (*p* = 0.0409) (Table 1; Figure 1B). Estimated lymphocyte counts varied significantly by year (Kruskal-Wallis ANOVA; *h* = 11.31, *p* = 0.0233), driven primarily by 2018 being lower than 2022 (*p* = 0.0199; Table 1; Figure 1C). Estimated monocyte counts varied significantly by year (Kruskal-Wallis ANOVA; *h* = 10.51, *p* = 0.0327), driven primarily by 2021 being lower than 2018 (*p* = 0.0403; Table 1; Figure 1D). Estimated basophil counts varied significantly by year (Kruskal-Wallis ANOVA; *h* = 35.93, *p* < 0.0001) (Figure 1E). Differences by year were driven by mean rank counts for 2017 being significantly higher in comparison to several other years: 2018 (*p* = 0.0486), 2021 (*p* < 0.0001), and 2022 (*p* < 0.0001), and 2021 being significantly lower than 2018 (*p* = 0.0231) and 2019 (*p* = 0.0488) (Table 1; Figure 1E).

**Table 1:** A representation of the values obtained for estimated white blood cells count comparisons by year.

Years	Est. Total White Blood Cell Count		Est. Heterophil Count		Est. Lymphocyte Count		Est. Monocyte Count		Est. Basophil Count	
	Mean rank difference	*p*-value	Mean rank difference	*p*-value	Mean rank difference	*p*-value	Mean rank difference	*p*-value	Mean rank difference	*p*-value
2017 vs. 2018	22.91	>0.9999	25.10	>0.9999	12.51	>0.9999	−11.51	>0.9999	46.01	0.0486
2017 vs. 2019	16.20	>0.9999	49.76	0.0409	−13.20	>0.9999	22.20	>0.9999	47.26	0.0627
2017 vs. 2021	49.49	0.0787	86.87	<0.0001	7.010	>0.9999	40.65	0.2904	101.2	<0.0001
2017 vs. 2022	−18.26	>0.9999	24.66	>0.9999	−38.65	0.2364	−0.7320	>0.9999	78.18	<0.0001
2018 vs. 2019	−6.705	>0.9999	24.66	>0.9999	−25.71	>0.9999	33.71	0.4480	1.251	>0.9999
2018 vs. 2021	26.59	>0.9999	61.77	0.0066	−5.497	>0.9999	52.16	0.0403	55.15	0.0231
2018 vs. 2022	−41.17	0.1285	−0.4321	>0.9999	−51.16	0.0199	10.78	>0.9999	32.17	0.5142
2019 vs. 2021	33.29	0.7979	37.11	0.5085	20.21	>0.9999	18.44	>0.9999	53.90	0.0448
2019 vs. 2022	−34.47	0.4880	−25.09	>0.9999	−25.45	>0.9999	−22.94	>0.9999	30.92	0.7655
2021 vs. 2022	−67.76	0.0031	−62.21	0.0093	−45.67	0.1503	−41.38	0.2753	−22.98	>0.9999

Comparisons were made in GraphPad Prism using a Kruskal-Wallis ANOVA with Dunn’s multiple comparisons analysis, comparing the mean ranks of the cell counts each year, giving the *p*-values showing the significance in the differences in the mean rank differences in estimated counts by year. Mean rank difference is the value obtained when the mean rank of the second year in the comparison is subtracted from the first year. A negative value for mean rank difference indicates that the mean rank in the second year in the comparison is larger than the first year and a positive value for mean rank difference indicates that the mean rank in the second year is smaller than the first year.

### 3.2 Estimated white blood cell count variation with sex, body condition index, nesting propensity, and *Leucocytozoon* burden

We found that estimated counts of several white blood cells varied significantly by sex, with males having significantly lower estimated total white blood cell, heterophil, and lymphocyte counts than females (*p* = 0.0009, 0.0009, and 0.0330) (Table 2). No relationship was found in estimated monocyte and basophil counts between the sexes during the sample period (Table 2). We found that estimated monocyte counts were strongly related to BCI, with more negative BCI values in individuals who had higher estimated monocyte counts (*p* = 0.0249) (Table 2). Estimated total white blood cell, heterophil, lymphocyte, and basophil counts, did not show a significant relationship when compared to BCI (Table 2). Murrelets that did not attempt to nest had significantly higher estimated lymphocyte counts than those that did nest, (*p* = 0.0328), however no correlations with total white blood cell count, heterophil count, monocyte count or basophil count were noted (Table 2). Murrelets with a higher *Leucocytozoon* burden had significantly lower estimated total white blood cell and heterophil counts (*p* = 0.0147 and 0.0358), but no changes in lymphocyte, monocyte or basophil counts (Table 2).

**Table 2:** A representation of the values obtained for estimated white blood cells count comparisons by sex, BCI, nesting attempt, and *L. marmoratii* burden after taking into account variations present by year and all other covariates using a multiple linear regression analysis with a quasi-Poisson distribution.

Est. White blood cell count ~ Year + Sex + Body condition index + Nesting(Y/N) + Burden	Est. Total White Blood Cell Count			Est. Heterophil Count			Est. Lymphocyte Count			Est. Monocyte Count			Est. Basophil Count		
	Estimate	Standard error	*p* value	Estimate	Standard error	*p* value	Estimate	Standard error	*p* value	Estimate	Standard error	*p* value	Estimate	Standard error	*p* value
Year	−0.0052	0.0187	0.7796	−0.0871	0.0248	0.0005	0.0585	0.0231	0.0120	−0.0647	0.0469	0.1686	−0.1768	0.0462	0.0002
Sex	−0.1622	0.0485	**0.0009**	−0.2102	−0.0628	**0.0009**	−0.1306	0.0610	**0.0330**	0.0565	0.1203	0.6389	0.0950	0.1132	0.4018
BCI	−0.0016	0.0011	0.1546	−0.0000	0.0014	0.9980	−0.0020	0.0014	0.1470	−0.0064	0.0029	**0.0249**	0.0002	0.0026	0.9419
Nesting	0.1258	0.0829	0.1299	0.0362	0.1052	0.7312	0.2296	0.1070	**0.0328**	0.1165	0.2092	0.5779	0.1242	0.1987	0.5324
Burden	−0.0039	0.0016	**0.0147**	−0.0042	0.0020	**0.0358**	−0.0036	0.0020	0.0789	−0.0011	0.0038	0.7798	−0.0058	0.00388	0.1343

The value for estimate shows the log odds of the correlation, giving a positive value for a positive relationship and a negative value for a negative relationship. The table also shows standard error and the *p*-value for the relationship. Bolded values are *p*-values less than 0.05.

### 3.3 Reference intervals

**Figure 2:**
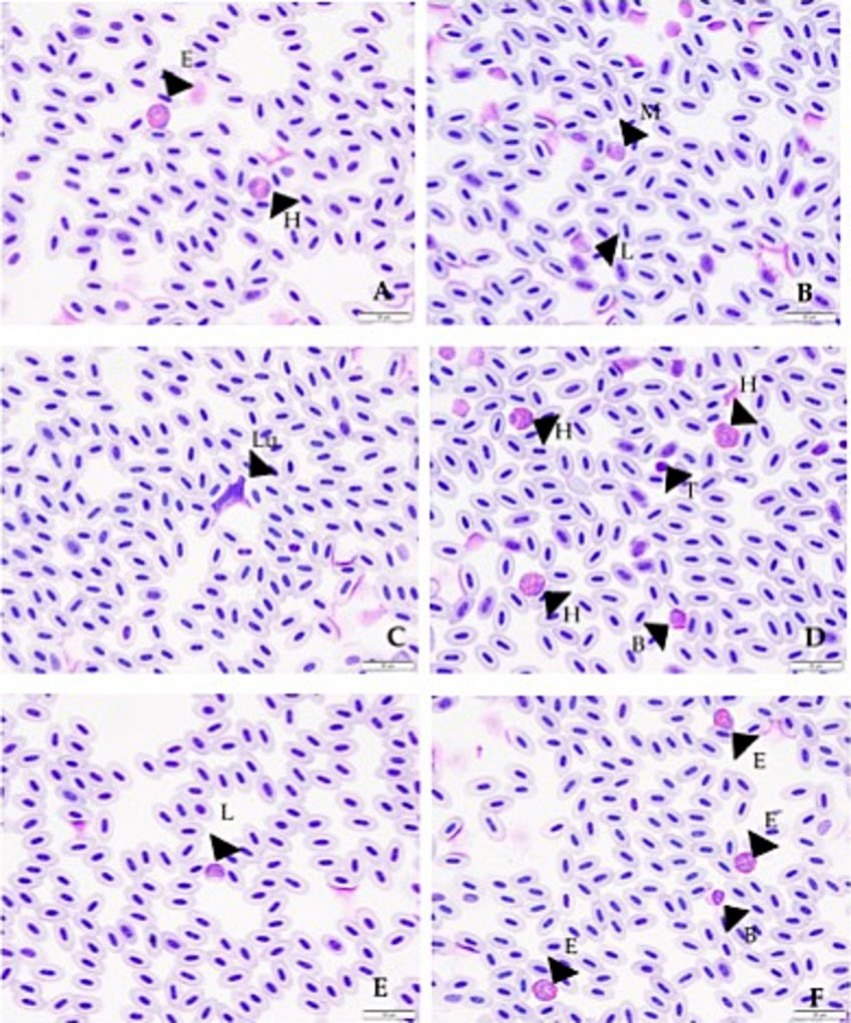
Photographs showing examples of the different white blood cells as found for Marbled Murrelets in this study. Each arrow with letter corresponds to a cell or other finding. **(A)** Arrow with E shows an eosinophil and arrow with H shows a heterophil. **(B)** Arrow with M shows a monocyte and arrow with L shows a lymphocyte. **(C)** Arrow with Lu shows a Leucocytozoon (*L. marmoratii*) within a cell. **(D)** Arrows with H’s show heterophils, arrow with B shows a basophil, and arrow with T shows a thrombocyte. **(E)** Arrow with L shows a lymphocyte. **(F)** Arrows with E’s show eosinophils and arrow with B shows basophil.

Reference Intervals were generated from blood samples taken from 130 murrelets that were clinically healthy and showed no *L. marmoratii* burden during 2017–2019 and 2021–2022, with the sample period being late April through early June of each year (Table 3). Table 3 shows the values obtained for estimated total white blood cell count, percent of each white blood cell type that makes up the total count, and the differential counts for each cell type. The 90% confidence intervals displayed in the table were calculated for the upper and lower limits of each reference interval. Examples of each type of cell are shown in Figure 2.

**Table 3:** A representation of the values obtained for estimated total white blood cell count, relative percent of each white blood cell type that makes up the total count, and the estimated differential counts for each cell type from *n* = 130 murrelets between 2017–2019 and 2021–2022.

Parameter	Mean	Median	IQR	Min	Max	Reference interval	90% Confidence interval of lower limit	90% Confidence interval of upper limit	*p*-value
Estimated Total WBC Count (10^9^/L)	9023.8	8250.0	6,750–11512.5	1800	20,600	3110.0–17,945	1800–3,800	1700–20,600	0.000
Relative Lymphocyte (%)	55.1	55.5	44–65.75	19	86	22.3–85.0	19.0–31.0	79.0–86.0	0.678
Relative Heterophil (%)	38.7	39.0	29.25–49	9	80	10.6–73.5	9.0–13.0	64.0–80.0	0.542
Relative Monocyte (%)	3.2	2.0	1.0–5.0	0.00.00	17	0.0–12.0	0.0–0.0	9.0–17.0	0.000
Relative Basophil (%)	2.7	2.0	1.0–4.0	0.0	11	0.0–9.7	0.0–0.0	7.0–11.0	0.000
Relative Eosinophil (%)	0.2	0.0	0.0–0.0	0.0	5	0.0–2.0	0.0–0.0	1.0–5.0	0.000
Estimated Lymphocyte (10^9^/L)	4956.33	4220.0	3,060–6112.5	954	16,274	1,116–12,907	954.0–1500.0	10370.0–16274.0	0.000
Estimated Heterophil (10^9^/L)	3404.5	3076.0	2248.5–4,281	270	10862.5	586–8,838	270.0–1178.0	7680.0–10,863	0.000
Estimated Monocyte (10^9^/L)	320.7	169.0	75.25–454	0.0	2,346	0–1,404	0.0–0.0	1072.0–2346.0	0.000
Estimated Basophil (10^9^/L)	231.6	170.0	73–363.5	0.0	972	0–742	0.0–0.0	675.0–972.0	0.000
Estimated Eosinophil (10^9^/L)	16.45	0.0	0.0–0.0	0.0	350	0–238	0.0–0.0	124.0–350.0	0.000

90% confidence intervals were calculated for the upper and lower limits of the reference interval using non-parametric methods in a macroinstruction from Microsoft Excel, Reference Value Advisor. The Reference Value Advisor was also used to acquire values for mean, median, inter-quartile range (IQR), maximum, and minimum using standard untransformed data, and *p*-value using the Anderson-Darling test for normality. The distribution and method for all reference intervals in the table are non-Gaussian (NG) and non-parametric (NP).

## 4 Discussion

Our study demonstrated the value of hematological analysis in addition to more traditionally collected covariates used in the study of seabirds, such as sex, body condition index, nesting propensity, and *L. marmoratii* burden, in the evaluation of individual and population health. We found that estimated white blood cell counts varied compared to several factors, inducing across years, and by sex, body condition index, nesting propensity, and *L. marmoratii* burden.

We found that estimated white blood cell counts varied by year in this study. Wild bird species respond to the seasonal changes in their environments and leukocyte profiles may reflect these changes throughout the year or over time (62). There are many additional factors that may influence white blood cell counts in wild avian species such as environmental contaminants, prey abundance, and habitat quality, all of which can affect immune function (63–66). This effect of stressors on immune function may result in changes in white blood cell counts and may also explain why we found that estimated white blood cell counts appear to vary across years. It is particularly intriguing that the granulocytes showed significant variation across all the years, suggesting they may be more responsive to environmental changes than the monocytes and lymphocytes – which would make them important to monitor in populations of interest. Additionally, in 2021 multiple white blood cell counts were significantly lower than in other years (Figure 1), perhaps indicating that the birds were suffering from reduced parasite and pathogen challenges that year, which is also the year in which our study population experienced the most favorable ocean conditions and exhibited the greater nesting propensity (37%, unpublished data, JWR) (34, 67). This suggests that murrelets may be subject to energy-based trade-offs between stressors, immune function, disease, and reproductive effort, as has been found for other species (68–70).

We noted a significant relationship between *Leucocytozoon* burden and estimated white blood cell counts where murrelets with a larger burden of *L. marmoratii* had a significant decrease in both estimated total white blood cell and estimated heterophil counts. At this time, the blood cell type(s) that *L. marmoratii* merozoites invade is unknown; however, we know that different species of *Leucocytozoon* can invade either red blood cells or mononuclear white blood cells such as monocytes and lymphocytes (71). Other species, including raptors, are often hosts for *Leucocytozoon* species, and experience decreased lymphocyte and monocyte counts and anemia, which is in contrast with our findings in murrelets (72, 73). The difference in the relationship we found between estimated white blood cell counts and parasite burden in murrelets compared with other species may indicate that the effect of parasitemia on murrelets is more driven by additional factors than those we investigated and that additional research on this topic is warranted. In particular, investigation as to the pathophysiology of *L. marmoratii* and murrelets would allow further characterization of the impact that parasitism may have on murrelets.

We noted differences between males and females when comparing sex to estimated white blood cell counts in this study. Estimated total white blood cell, heterophil, and lymphocyte counts were significantly higher for female birds compared to male birds, during the sample period. This is perhaps not surprising, as a recent meta-analysis suggested that seasonal and sex differences in the immune system may be common in birds in general, and that differences between males and females appear to be stronger during the breeding period than in other times of the year (74). Nevertheless, it is difficult to interpret the reason why estimated white blood cell counts differed between males and females in our study; however, we can speculate based upon our knowledge of white blood cell function and the conditions that may cause a change in their relative numbers. In general, a decrease in lymphocyte counts may be related to a reduction in immune system energy allocation and function associated with stressors, and can vary in number based upon immune response in the identification and response to pathogens (75). It seems unlikely that the difference in estimated mean white blood cell counts between the sexes that we observed is due to a pathogen, as we would expect both sexes to be affected equally if that was the case. Related findings were published by Ots and Hõrak in 1998 during their study of sex-specific clinical profiles of Great Tits (*Parus major*) (76). In this study, male tits showed lower lymphocyte counts in the breeding season, which was speculated to be a result of the increase in testosterone and corticosterone hormones that are present in the breeding period for this species. Additionally, in the Little Auk (*Alle alle*), another small seabird species in the family Alcidae that has a very similar breeding ecology as murrelets (i.e., monogamous, shared incubation), it was demonstrated that males had lower lymphocyte counts in the early incubation period compared to females (77). Behavioral observations of Little Auks in this study showed that, while males and females appeared to share incubation duties on the nest equally, that males appeared to be engaging in other activities other than foraging while they were off the nest, including aggressive interactions with other members of the colony. These social interactions may explain the differences in white blood cell counts in male Little Auks vs. females in the early incubation phase (77). Murrelets are non-colonial, and it is unknown if males engage in territorial behavior when off the nest at this time (15). However, it is possible that at this time of year during the breeding season and before egg laying, male murrelets are experiencing stressors that are apparent through changes in white blood cell counts compared to females, that we do not understand at this time.

We found that as BCI decreased, estimated lymphocyte counts and monocyte counts increased. Lymphocytosis, the relative increase in quantity of lymphocytes in the peripheral blood in birds, can be caused by infections that cause antigenic stimulation, such as viral infections (75). Monocytosis, the relative increase in quantity of monocytes in the peripheral blood in birds, can be caused by chronic diseases such as bacterial or fungal disease, or in some cases associated with mineral deficient diets (75). Immune system stimulation can be energetically expensive and may cause a decrease in fitness leading to decreased body condition (78). At this time, the exact mechanism explaining the correlation between white blood cell counts and body condition index for murrelets remains unknown. However, these findings further support the use of the white blood cell counts in the field of wildlife research, as the association between BCI and estimated white blood cell count elevations may correlate to the development of disease within a population or an individual, warranting further investigation as to the cause.

We found that nesting propensity was significantly correlated to estimated lymphocyte counts; individuals that were less likely to attempt to nest tended to have significantly higher estimated lymphocyte counts than birds that did attempt to nest. It has been found in other seabird species that during the pre-laying and laying phases of the breeding period the stress and effort associated with breeding may cause alterations in white blood cell counts, namely a decrease in lymphocytes and an increase in heterophils (74, 75, 79, 80). Our modeling accounted for several covariates, including sex; therefore, this decrease in estimated lymphocytes appears to be affecting both sexes. It is possible that this decrease in estimated lymphocytes in birds more likely to attempt a nest, seen in both sexes, could be attributed to changing hormone levels or stressors during the breeding season associated with nesting, and that birds that are not nesting are not experiencing these changes in hormone levels or other stressors associated with breeding to as great of an extent, maintaining their lymphocyte counts at higher levels (81–84). During the time of year that our sampling occurred, murrelets were likely in the pre-laying and/or early laying period of their breeding season (32) therefore, the effect of hormones or the energetic costs of egg laying in the female could be causing the decrease in estimated lymphocyte counts we are seeing (79).

A separate published study, conducted in Alaska and published in 1997 by Newman et al., sought to publish reference intervals for a variety of seabird species, including the murrelet population in that area (9). In that study Marbled Murrelets were sampled, and samples were collected in June of 1990 from the Shumigan Islands, Alaska. The mean counts derived from these samples appear to generally fall within the reference intervals that were obtained in this study and were collected during a similar time of year; however, the sample sizes used for analysis varied widely between the two studies (*n* = 11 by Newman et al. (9), and *n* = 130 in this study). Although these two studies were conducted in two separate geographical areas, comparison of reference intervals within the same species over time is a helpful tool in identifying trends and changes in population health that may be developing. This is because the study of blood from living animals over time provides an additional benefit in proactive management of individual or population health, rather than reactive management in response to a health event, such as high mortality or failure of recruitment (9). When estimating reference intervals, the larger the sample size, the less degree of uncertainty and the greater the likelihood that the sample is representative of the population as a whole (11). Thus, the large sample analyzed in our study is beneficial in providing a large number of specimens to produce statistically significant reference intervals that are representative of the population we have investigated.

## 5 Conclusion

We found that changes in estimated white blood cell counts can be correlated to morphologic, seasonal, infectious, and external factors. Importantly, we found that estimated white blood cell counts varied by year with several parameters being significantly lower in 2021, the year that was found to have the best ocean conditions and nesting propensity compared to other years. It would be valuable to evaluate white blood cell counts across a wide range of ocean conditions to better understand the drivers of this pattern. This study also established reference intervals for white blood cell count estimates for the Oregon population of the Marbled Murrelet. These reference intervals can provide information about the health of this population and can be used over time to look for deviations that may be associated with challenges affecting the species. The relationship between changing environmental conditions and white blood cell parameters is significant from a conservation standpoint, because poor and variable ocean conditions are expected to increase in the future, and the monitoring of blood values over time can help us further understand the immunological effects that these changes are having on murrelets and other seabird species.

## Data Availability

The datasets presented in this study can be found in online repositories. The names of the repository/repositories and accession number(s) can be found in the article/supplementary material.
